# Winter is (not) coming: Acoustic monitoring and temperature variation across important bat hibernacula

**DOI:** 10.3897/BDJ.13.e141801

**Published:** 2025-01-24

**Authors:** Nia Toshkova, Maksim Kolev, Stanimira Deleva, Tzvetan Simeonov, Vasil Popov

**Affiliations:** 1 National Museum of Natural History at the Bulgarian Academy of Sciences, Sofia, Bulgaria National Museum of Natural History at the Bulgarian Academy of Sciences Sofia Bulgaria; 2 Institute of Biodiversity and Ecosystem Research at the Bulgarian Academy of Sciences, Sofia, Bulgaria Institute of Biodiversity and Ecosystem Research at the Bulgarian Academy of Sciences Sofia Bulgaria; 3 Meteorological Observatory Lindenberg, Deutscher Wetterdienst (DWD), Lindenberg, Germany Meteorological Observatory Lindenberg, Deutscher Wetterdienst (DWD) Lindenberg Germany

**Keywords:** winter activity, acoustic monitoring, bats, hibernation, climate change, behaviour, caves

## Abstract

Little is known about the winter bat activity in Bulgaria, which poses challenges in monitoring potential deviations in their behaviour as a consequence of the warming climate. Using passive acoustic monitoring, we investigated the winter activity in some of Europe’s largest hibernacula. Our findings reveal cave and species-specific activity patterns. Activity was observed throughout each month of the survey, with distinct peaks on specific days. At one high-elevation site, bat activity was restricted to a single night, while the highest overall activity occurred at the highest elevation site (1325 m). The most active species was *Myotiscapaccinii* (Bonaparte, 1837). While bats were mostly active right after sunset following their usual circadian rhythm, some daytime activity was also observed, including emergence at temperatures as low as -8°C. At sites with sufficient activity data, external temperature emerged as a significant positive predictor of bat activity, with higher temperatures associated with increased activity. Our data also suggest that bats rarely forage near the roost entrances. The observed variability in activity levels between study sites highlights the need for high-resolution, site-specific data rather than broad generalisations.

## Introduction

Bats are a diverse and ecologically important group of mammals. However, various factors, such as habitat loss, disturbance, disease and climate change, threaten their well-being (Frick et al. 2019). Understanding how bats cope with these challenges, especially during vulnerable periods of their life cycle, is pivotal for their effective conservation. Hibernation is a crucial aspect of bat biology in temperate regions. It involves extended periods of torpor during which bats lower their body temperature and metabolic rates to cope with scarce resources (Geiser and Ruf 1995). During this vulnerable period, caves provide stable microclimatic conditions that protect them from inclement weather and reduce the loss of body water (Kunz 1982, Avila-Flores and Medellín 2004). A natural part of hibernation is the periodic arousal from torpor, which is associated with water, food, sleep and immune system maintenance requirements (Speakman and Racey 1989, Thomas 1995, Luis et al. 2006). However, the frequency and duration of these arousals vary depending on the species, their hibernacula, local weather and food availability (Wojciechowski et al. 2007, Zahn and Kriner 2016, Barros et al. 2017, López-Roig et al. 2024).

The winter activity of bats affects their energy use, health and ultimately their survival (Boyles et al. 2006). This became evident since the emergence of the White-nose disease (WND) which causes mass mortality events in hibernating North American bats (Frick et al. 2010). The disease leads to abnormal winter activity and energy depletion in the infected animals (Reeder et al. 2012). Likewise, temperature fluctuation and extreme weather events during the winter, such as heatwaves, floods and prolonged cold snaps, may also lead to abnormal winter activity (O'Shea et al. 2016). The consequences of this could be complex, affecting various species and populations differently (Reusch et al. 2019, Reusch et al. 2023). Some species may benefit from milder winters that provide feeding opportunities (Bernard et al. 2021, Toshkova et al. 2023), while others may suffer adverse effects due to increased mortality risks and direct impacts on fitness (Reusch et al. 2019, Salguero et al. 2023). However, direct evidence and long-term studies are rare for most mammalian hibernators (Findlay‐Robinson et al. 2023). In some regions, such as the Mediterranean, predictive models suggest that bats may cease hibernation by the end of the century (Mas et al. 2022). Longitudinal data have shown a consistent decline in the pre-hibernation body weight of *Miniopterusschreibersii* (Kuhl, 1817) in Spain over the last decades (López-Roig et al. 2024). Consequently, individuals are entering the hibernation period without a sufficient increase in their energy reserves, possibly as adaptations to milder winters. However, extended periods of cold snaps might pose an increased threat to this species. Therefore, it is essential to gain a better understanding of the risk factors associated with increased winter activity and how different species and populations adapt.

The Balkan Peninsula is a biodiversity hotspot (KryŠtufek 2004) and Bulgaria, in particular, hosts some of Europe's largest hibernation colonies (Ivanova 2005, Deleva et al. 2023). However, data on bat hibernation have only been sporadically published and only two studies to some extent examine the winter activity, focusing on the winter diet and the year-round bat activity in an urban environment (Toshkova et al. 2023, Kolev et al. 2024). To fill in this gap, we conducted passive acoustic monitoring at the roost entrance of four underground hibernacula. We hypothesised that: (1) cave temperatures where bats hibernate will not be affected by the external temperature; (2) bats in high-altitudinal caves will experience more stable external winter conditions and will exhibit lower activity levels than bats at low-altitudinal caves and (3) fluctuations in ambient temperature would positively affect the activity patterns.

## Materials and methods

**Studied sites and species**

**Figure 1.**
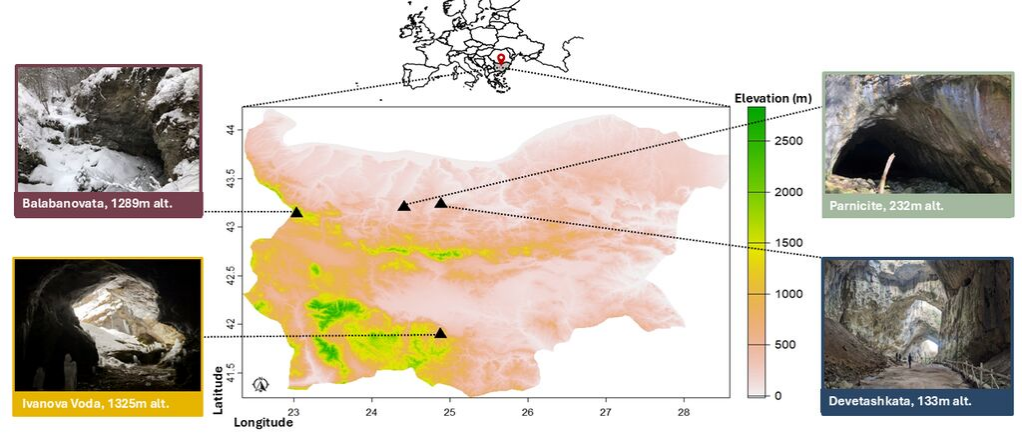
Geographic locations (black triangles) of the study sites on a topographic map of Bulgaria.

We conducted our research in four underground sites (Fig. 1), identified as bat hibernacula of international importance (Ivanova 2005, Deleva et al. 2023). All of the sites have high Biotic Potential (BP = 1) and varying levels of roost vulnerability to anthropogenic disturbance - BV index A, B and C, according to the Bat Cave Vulnerability Index (see Deleva et al. (2023)). We sought overlaps in species composition, colony sizes and altitudes corresponding to the species' preference for hibernation, as well as similarities in the presence of the causative agent of WND, the fungus *Pseudogymnoascusdestructans* (Minnis & Lindner, 2013) (Table 1).

Golyamata Balabanova cave (Balabanovata) is located within “Zapadna Stara Planina I Predbalkan (BG0001040)” Natura 2000 protected zone designated under the EU Habitats Directive (Council Directive 92/43/EEC). During the winter period *Myotismyotis* (Borkhausen, 1797) and *Myotisblythii* (Tomes, 1857) aggregate in the cave. Ivanova Voda is located within the Natura 2000 zone “Rodopi - Sredni” (BG0001031). It provides a roost for eight bat species year-round, three of which hibernate there. Both of these roosts are affected by the WND and host around 90% of the known hibernating populations of *Myotismyotis* and *Myotisblythii* in Bulgaria (Petrov 2010, Petrov 2015, Toshkova and Deleva 2022). The population sizes of the two mixed colonies of *M.myotis*/*blythii* are currently identical, with 2500 in Balabanovata and 2600 individuals roosting in Ivanova Voda (Toshkova and Deleva 2022). Balabanovata was recently included in the national monitoring scheme, so only historical data from Ivanova Voda are available, indicating a decline in *M.myotis*/*blythii* numbers, consistent across other species that hibernate in the cave, like the long-fingered bat (*Myotiscapaccinii*) (Petrov 2010, Petrov 2015, Toshkova and Deleva 2022). To our knowledge, this site is also the highest known hibernaculum for the species (Benda et al. 2003, Aihartza and Aizpurua 2023).

Devetashka Peshtera (Devetashkata) is a low-elevation cave, located within the designated protected area known as "Devetashkata Peshtera" and is classified as a Natural Landmark. This cave hosts 17 bat species year-round and three of them use the roost during hibernation, forming colonies of tens of thousands of individuals (Ivanova 2005, Toshkova and Deleva 2022, Deleva et al. 2023). The numbers varied between the years as most probably those colonies also utilised several other hibernacula in the region. Amongst them is the largest known *Nyctalusnoctula* (Schreber, 1774) colony in the country. Devetashkata is recognised as one of the 100 national tourist sites and has experienced increasing tourist activity since 2011, leading to growing anthropogenic pressure (Ivanova 2017). The other low-elevation cave, Parnicite Dolen Parnik (Parnicite), is protected within the “Dolniya Parnik-Peshtera” protected area and is classified as a National Landmark. Five bat species are known to inhabit the cave year-round, with three species aggregating during the winter, forming colonies of more than 60,000 bats (Toshkova and Deleva 2022). However, declining numbers of *Myotiscapaccinii* using Parnicite have also been observed, suggesting changes in the suitability of the cave as a hibernaculum for this species or even potential population decline (Petrov 2010, Petrov 2015, Toshkova and Deleva 2022).

**Figure 2.**
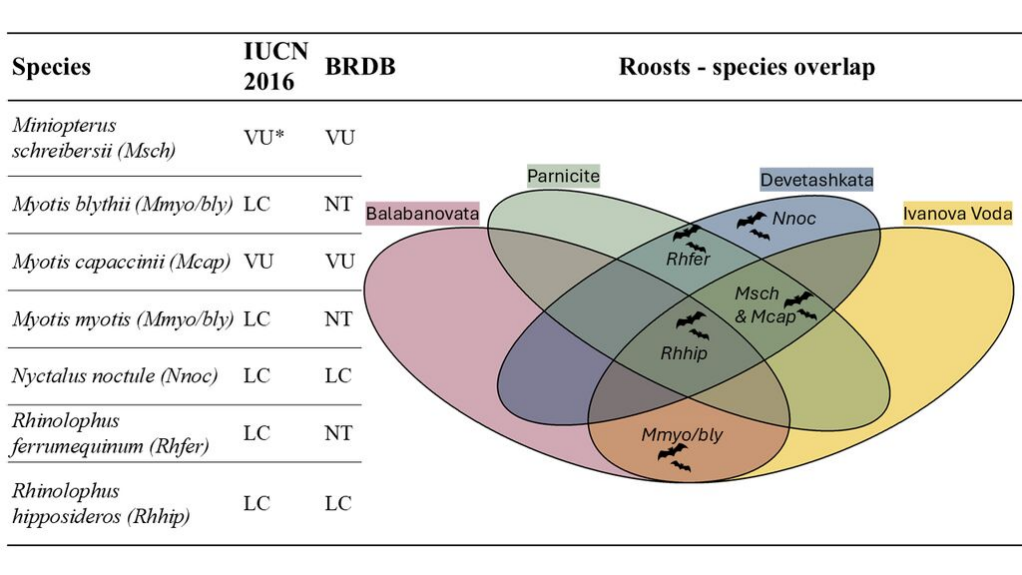
List of bat species hibernating in the study sites, including their conservation status according to the global assessment (IUCN 2016; IUCN 2023*), their local assessment (BRDB = Bulgarian Red Data Book 2015) and a Venn diagram presenting the overlaps between the species compositions in the study sites.

All bat species that hibernate in the studied caves (Fig. 2) are protected under the Bulgarian Biological Diversity Act and listed under various agreements and conventions, including the European Agreement for the Conservation of Bat Populations (EUROBATS), the Berne Convention on the Conservation of European Wildlife and Natural Habitats and the BON: Appendices of the Convention on the Conservation of Migratory Species of Wild Animals.

**Hibernacula survey and acoustic monitoring**

We collected bat census data from each study site in 2022, immediately before initiating the passive acoustic monitoring, in order to record the species composition and quantities. We conducted visual assessments directly within the roosts and photographed the colonies. This approach enabled precise scoring of individuals or estimation of the colony size using the ratio between surface area and the bat aggregation densities (Deleva 2023). As an addition to our study, we placed a guano trap (as in Toshkova et al. (2023)) beneath the largest bat cluster to monitor faecal accumulation as an indicator of feeding activity. The guano traps in all but Balabanovata cave were displaced by water, resulting in relevant data on guano accumulation from only one site.

We acoustically monitored the cave-exiting activity in the selected hibernacula. Using Anabat Swift (Title Scientific, UK), we recorded bat activity at the entrances of Ivanova Voda, Parnicite and Balabanovata, while Audiomoth (Hill et al. 2018) was utilised at the entrance of Devetashkata cave. At Parnicite and Balabanovata, the detectors were positioned one metre from the entrances, pointing perpendicular to the bats' most probable exiting route. For Ivanova Voda, the detector was situated at the top of the roost, three metres from the entrance. Devetashkata has one main entrance and several large openings in the ceiling preceding it. The main entrance of the cave is notably large (measuring 30 х 35 m in height and width), with instances of detectors being stolen from there in the past. Consequently, we deployed the low-cost detector (Audiomoth) at one of the vertical openings where mist netting is typically conducted and where there is no tourist trail. We recorded bat activity throughout the entire 24-hour period during the winter months (from January to March) of 2022, with the recording duration varying for each roost. In total, recordings were made for 196 days.

**Sound identification**

Bat echolocation calls were recorded as full-spectrum calls in WAC format and later converted to WAV using Kaleidoscope software (version 5.6.6, Wildlife Acoustics, Inc., Maynard, MA, USA). Bat passes (i.e. a sequence of ≥ 2 bat calls in zero-crossing (ZC)) were аutomatically separated into files with a maximum duration of five seconds. Bat activity was measured by counting the number of 5-second sound recordings that contained at least two echolocation calls. We visually identified search phase calls to species using the sound identification keys from Russ (2021) and Froidevaux et al. (2023). Measurements were collected for at least one representative call from each call sequence. We collected data on six acoustic parameters: the frequency of maximum energy (Fpeak), the frequency at the start of the call (Fstart), the frequency where the call ends (Fend), the duration in milliseconds (ms) between the start and end frequencies of the call (Dur), the duration in ms between calls (TBC) for all species and the slope of the body of the call (Sc) for FC-QCF bat calls. Those variables were collected using the automatic analyse view option in Kaleidoscope. Kaleidoscope settings were as follows: FFT size 512, window size 128 and cache size 256 MB. Representative bat calls were archived in the ChiroVox acoustic database (Görföl et al. 2022), securing independent validation of the assigned identifications and, crucially, promoting bat acoustic research within the realm of open science. A total of 279 calls (catalogued from A005833 to A006111) were deposited on 29 October 2024 and are freely accessible for download at the following link: ChirovoxUID. The dataset used in the analyses is included in Suppl. material 1.

**Weather data**

We acquired weather data including maximum daily temperatures, аverage daily temperature and temperature at sunset. The data were extracted from the European Center for Medium-Range Weather Forecast (ECMWF) fifth-generation Reanalysis (ERA5). The ERA5 is a global re-analysis of weather data, spanning from 1940 to the present, with assimilated data from the surface, upper-air and satellite observations with a spatial resolution of 0.1 degrees (10 km) and temporal resolution of 1 hour (Muñoz Sabater 2019). The data were extracted as temperature and daily accumulated precipitation from the surface ERA5-L and datasets with coordinates (Ivanova Voda N41.8629 E24.688, 1333 alt.; Balabanovata N43.1283 E23.07, 1287 alt.; Parnicite N43.1986 E24.4478, 224 alt. & Devetashkata N43.1986 E24.7164, 110 alt.) of the selected nods (Hersbach et al. 2023, Zippenfenig 2023). From these datasets, mean daily temperature was calculated and used in this study. Additionally, we recorded the cave temperature with temperature and humidity loggers iButtons (model DS1923-F5, Maxim Integrated Products, Inc., Sunnyvale, California) situated close to the largest colony in each hibernaculum. They were set to record air temperature twice daily (at 00:00 and 12:00 h) for the whole study period and mean daily temperature was calculated and used for further analyses. Precise temperature data at the time of bat activity were also obtained for three of the roosts (Ivanova Voda, Parnicite and Balabanovata) using the Anabat Swift's built-in temperature loggers. All the weather data used in the analyses can be found in Suppl. materials 2, 3.

**Statistical analysis**

All data visualisation, exploratory data analysis and models were performed in R (version 4.1.1). Using *MASS* and *pscl* packages in R (Venables and Ripley 2002, Zeileis et al. 2008), we tested different model fits. Based on the Akaike's Information Criterion (AIC; Burnham and Anderson (2004)), we selected zero-inflated negative binomial regression to assess the influence of temperature on bat activity at the two study sites with sufficient activity data (Parnicite cave and Ivanova Voda cave), with external and internal temperatures as predictors of bat activity patterns. To test the correlation between the external average daily temperature and the average cave temperature where the highest proportion of bats are hibernating in each study site, we employed linear regression models by applying the “lm” function in R. Historical temperature trends were also analysed using linear regressions. Maps were plotted using the following R packages “rnaturalearth” (Massicotte and South 2023), “elevatr” (Hollister 2017, Hollister 2023), “terra” (Hijmans 2023) and “sp” (Pebesma and Bivand 2005). The script used for the analysis is available upon request.

## Results

We recorded a total of 11,954 bat call sequences over 196 detector days across all locations. Table 2 provides details of the acoustic survey and census data, including the size and species composition of winter bat colonies at the study sites. An approximate estimate of the winter colony of *Nyctalusnoctula* in Devetashkata cave is also included, as direct monitoring was not feasible due to the inaccessible location.

Levels of bat activity differed between sites. In Devetashkata cave, bat activity was documented during all survey days (n = 9), with a total of seven species/acoustic groups and 171 call sequences recorded. In contrast, Balabanovata cave exhibited very low bat activity, with only a single night of detection (25/02/2022) involving *Rhinolophushipposideros* (Bechstein, 1800), a species that occasionally inhabits the cave. No activity of the most abundant resident species, *Myotismyotis*/*blythii* (2500 individuals), was detected at the entrance. Additionally, the guano trap in Balabanovata cave had no accumulation of faecal mater. Ivanova Voda cave recorded the highest bat activity, with 29 nights of activity and a total of 10,568 bat call sequences. Parnicite cave followed, with seven nights of activity and 1,190 bat call sequences recorded.

The most frequently recorded species was *Myotiscapaccinii*, accounting for over 69% of all recordings. It was followed by the *Myotismyotis*/*blythii* acoustic group (16.3%), *Miniopterusschreibersii* (1.7%), *Nyctalusnoctula* (1.1%) and *Rhinolophusferrumequinum* (Schreber, 1774) (0.9%). Other bat species were recorded with significantly lower activity (see Table 3). Notably, some species observed during the initial census were not actively exiting their roosts during the survey period.

**Figure 3.**
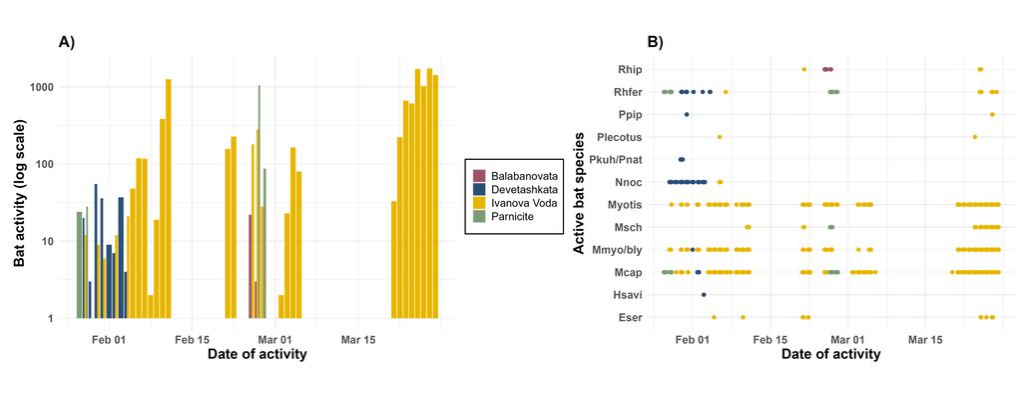
**A** Total activity patterns over the study period in each roost. Bat activity was measured by the number of sound recordings containing at least two echolocation calls; **B** Species-specific activity patterns for each roost. Species abbreviations from the y-axis are provided in the captions of Table 4.

**Figure 4.**
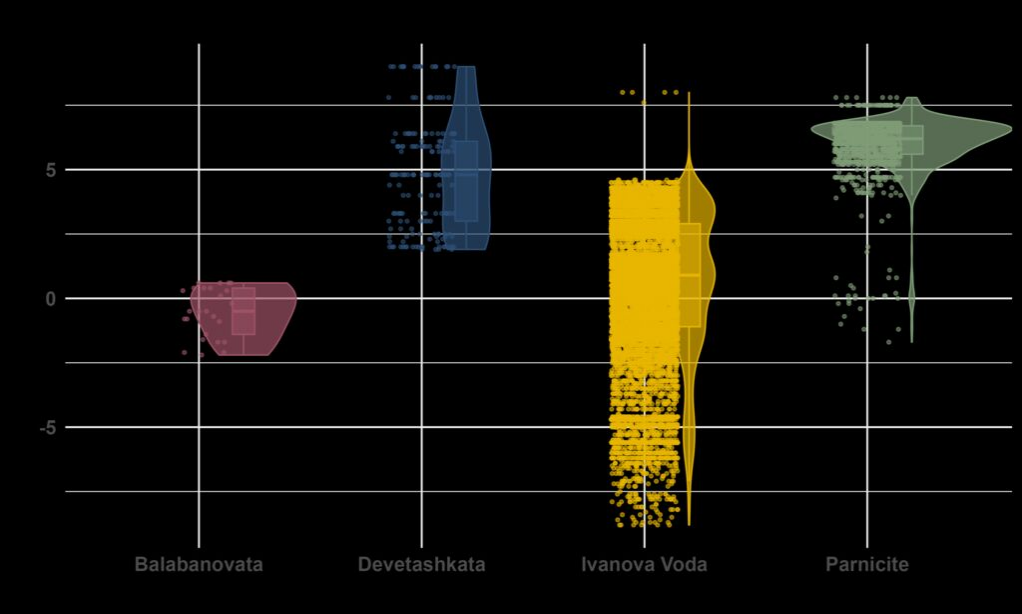
External temperature during bat activity at each study site. Each site is represented by a different colour and these colours are consistent across all figures.

**Figure 5.**
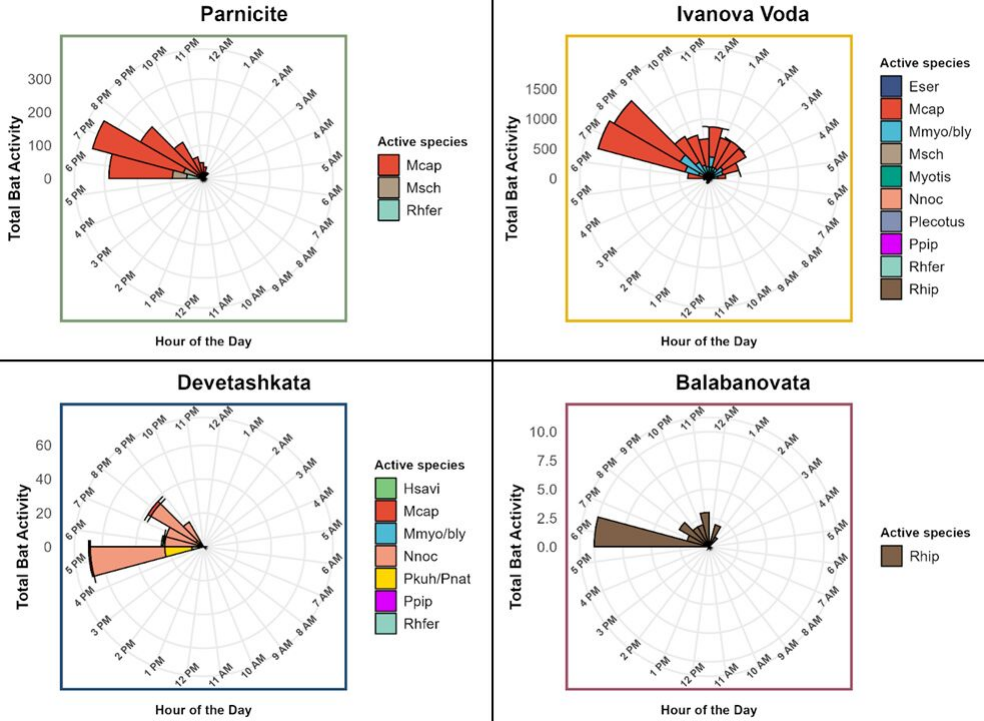
The 24-hour activity patterns of bat species in the four study roosts, represented using rose plots. Each plot illustrates the total species activity at each hour of the day. The x-axis indicates the time of day in a 12-hour AM/PM format, while the y-axis shows the bat activity for that period, measured by the total number of sound recordings containing at least two echolocation calls. Each species is represented by a different colour, as indicated in the legends. Species abbreviations in the legends can be found in the captions of Table 4.

Bats were active outside their winter roosts each month (January, February and March) at Ivanova Voda and Parnicite (Fig. 3). Peaks in activity were observed on specific days at both sites. In Parnicite, the peak of activity was in February, while in Ivanova Voda, there were peaks of activity in February and activity substantially increased towards the end of the hibernation period from mid-March onwards. The activity generally followed the typical circadian rhythms of bats and occurred at temperatures ranging from -8.8 to 8°C (Fig. 4 and Fig. 5). Bats were more active at warmer temperatures, but the temperature threshold for winter activity varied between roosts. Social calls and feeding buzzes were recorded only at the entrance of Ivanova Voda cave, which had the highest activity levels amongst the study sites. Bat activity was primarily observed after sunset until 02:00 h, although occasional diurnal activity (around noon) in Ivanova Voda cave was also recorded (Fig. 5).

**Figure 6.**
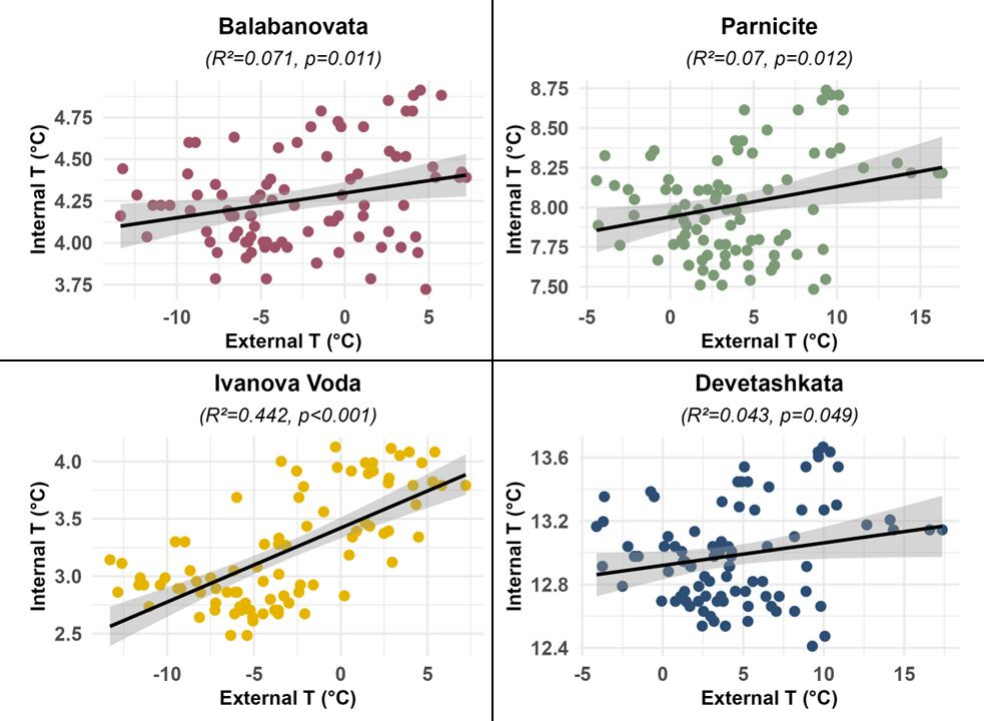
Correlation between external average daily temperature and internal cave temperature across study sites.

The external and internal temperature variations at each roost entrance and their summary statistics are shown in Table 4. The correlation between the external average daily temperature and the average internal cave temperature (at the location where the bats hibernate) varied across the study sites (Fig. 6). The R-squared (R²) values, which represent the proportion of variance in internal cave temperature explained by external temperature, were as follows: Balabanovata cave (R² = 0.07), Parnicite cave (R² = 0.07), Ivanova Voda cave (R² = 0.44) and Devetashkata cave (R² = 0.04). Specifically, R² values below 0.1 for Balabanovata, Parnicite and Devetashkata caves suggest no significant correlation between external and internal temperatures at these sites. In contrast, the R² value of 0.44 for Ivanova Voda cave indicates a significant contribution of external temperature to the variance in internal cave temperature, although it is not the sole influencing factor. Table 5 summarises the number of days during the winter of 2022 in each location when the external temperature at sunset and the mean daily temperature exceeded specific thresholds, highlighting periods of milder conditions across the sites. It can be clearly observed that the caves with higher entrance elevations have a lower number of days when the temperature at sunset is above 0°C.

**Figure 7.**
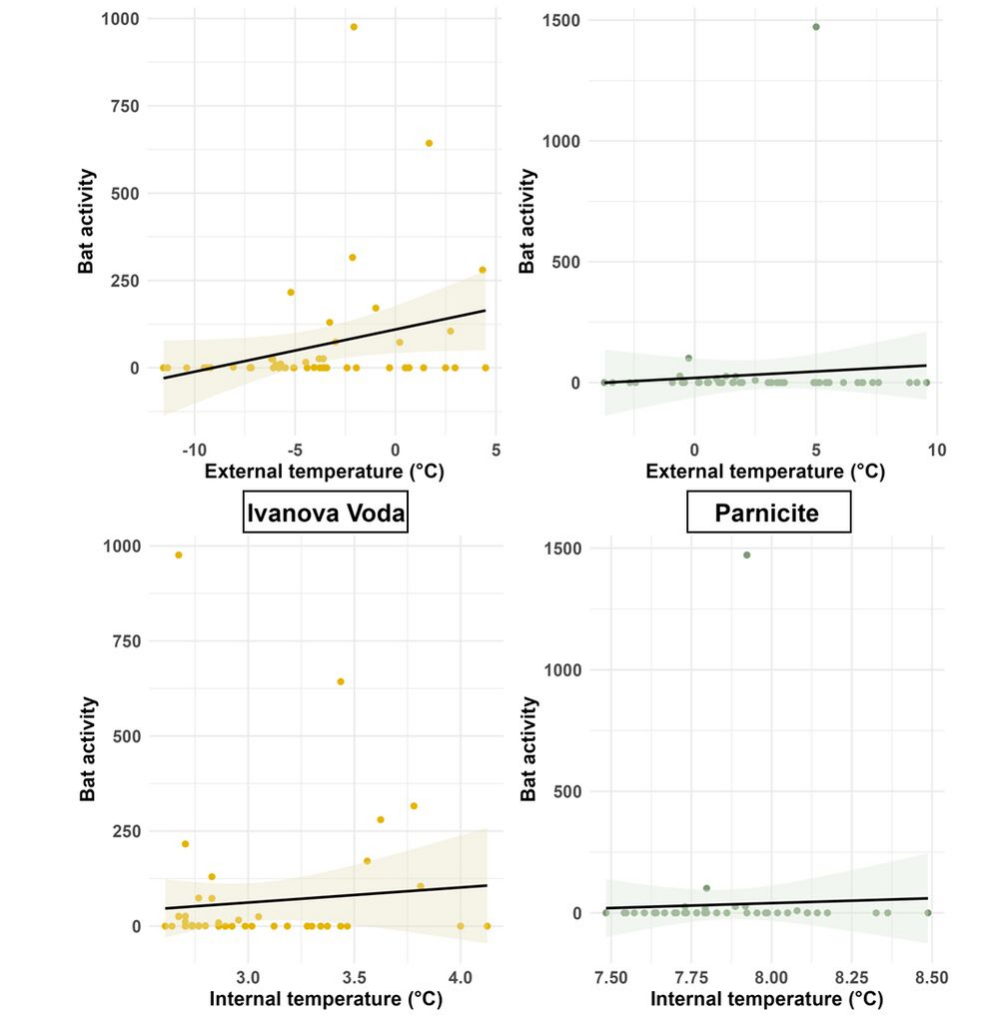
Effect of external and internal temperature on the activity pattern in Ivanova Voda and Parnicite. The bat activity represents the number of sound recordings containing at least two echolocation calls.

To assess the influence of temperature on bat activity at the two study sites with sufficient activity data, Parnicite cave and Ivanova Voda cave (Fig. 7), we fitted zero-inflated negative binomial regression using external and internal temperature as predictors for bat activity, with the zero-inflation component modelling the occurrence of zero counts (i.e. no bat activity). For Ivanova Voda, the model revealed significant relationships between external temperature and bat activity. The count model showed that an increase in external temperature was positively associated with bat activity (coefficient = 0.64, SE = 0.2, z = 2.9, p = 0.003). The internal temperature did not significantly affect activity (estimate = -1.03, z = -1.15, p = 0.24). The zero-inflation model did not reveal significant predictors for the occurrence of lack of bat activity. Both external (estimate = -0.017, z = -0.105, p = 0.9) and internal temperatures (estimate = 1.08, z = 0.92, p = 0.3) were not significantly related to the likelihood of zero activity. For Parnicite, the model also indicated a significant effect of temperature on bat activity. External temperature was significantly positively associated with activity (estimate = 0.84, z = 3.77, p = 0.0001), while internal temperature had a significant negative effect (estimate = -16.31, z = -2.4, p = 0.01). The zero-inflation model for Parnicite showed no significant predictors for zero counts. Both external (estimate = 0.4495, z = 1.405, p = 0.160) and internal temperatures (estimate = -5.0854, z = -0.896, p = 0.370) were not significantly related to the likelihood of no bat activity.

**Figure 8.**
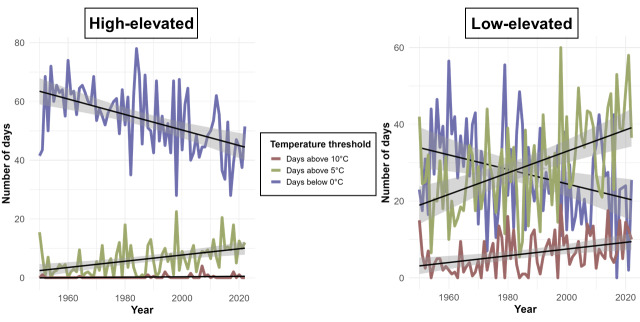
Historical temperature trends for the high-elevated (Ivanova Voda and Balabanovata) and low-elevated (Parnicite and Devetashkata) study sites, presenting the amounts of days with temperatures above specific thresholds considered important for bat activity. The amount of days with temperatures below freezing are decreasing, while the number of days with temperatures above 5 and 10 °C are increasing.

We analysed historical temperature trends for the external averaged daily temperature at our study sites from 1950 to 2022. For the purpose of this analysis, the caves were separated into two groups: high-elevation (Ivanova Voda and Balabanovata), which experience lower atmospheric temperatures and low-elevation (Parnicite and Devetashkata), which have higher atmospheric temperatures and similar trends. The results indicate a significant decrease in the number of days with temperatures below 0°C (p = 0.000005, R² = 0.25), with a decline of 2.5–2.7 days per decade for the higher-elevated caves, compared to 1.9–2.1 days per decade for the lower-elevated caves (p = 0.004, R² = 0.1). This suggests a shift towards milder winter conditions over time (Fig. 8). For days above 5°C, the trends indicate an increase of 1.2–1.4 days per decade for the higher-elevated caves (p = 0.0001, R² = 0.18) and 2.6–2.8 days per decade for the lower-elevated caves (p = 0.00001, R² = 0.24) . A smaller, yet equally significant rise in the number of days above 10°C was observed at the lower-elevated caves, with a trend of approximately 1.0 day per decade, indicating a broader trend towards warmer conditions (p = 0.001, R² = 0.14). The higher-elevated caves have very few days (between 0 and 5 days per year) with temperatures above 10°C during the months of January to March, which limits the confidence in the trend analysis of this data (R² = 0.06).

## Discussion

Winter bat activity studies have gained considerable popularity amongst researchers seeking to understand the ecological dynamics and behavioural adaptations of different species and populations during the colder months (e.g. Boyles et al. (2006), Halsall et al. (2012), Ben-Hamo et al. (2013), Hope and Jones (2013), Barros et al. (2017), Bernard et al. (2021), Czenze et al. (2017), Mas et al. (2022), Andersen et al. (2023), López-Roig et al. (2024)). However, no comprehensive study has yet addressed winter activity patterns across significant hibernation sites in many temperate regions, including Bulgaria. Our field-based approach allowed us to investigate these patterns across several of Europe’s largest hibernacula, providing crucial insights into the winter behaviour of bats in this region. Our findings reveal considerable variability in bat activity, highlighting the heterogeneous nature of activity across surveyed sites. The results underscore the importance of site-specific data for understanding bat ecology, especially as climate change progresses. Monitoring these activity patterns will be vital for assessing potential shifts in behaviour, identifying the complex ecological factors at play and implementing effective conservation strategies.

### Winter bat activity

Despite being located at a lower altitude and hosting a much larger number of bats, Parnicite cave showed less activity than Ivanova Voda. Although sampling efforts varied slightly during the study period, bat activity remained consistently higher in Ivanova Voda — even when excluding the extra survey days conducted there. The total number of bats in Ivanova Voda during the survey year was approximately 7,000, while Parnicite hosted a much larger population of 56,640 bats. This difference in activity levels suggests that factors beyond average external temperatures — such as hibernaculum suitability, fat reserves, predation pressure and foraging strategies — may influence the activity patterns observed between the sites.

Our findings indicate that *Myotiscapaccinii* exhibited the highest activity levels, despite being smaller than both *Myotismyotis* and *Myotisblythii*, as well as *Rhinolophusferrumequinum*. This observation challenges the expected trend, where larger bats are generally predicted to spend less energy in maintaining their body temperature and to be more active at low ambient temperatures compared to smaller bats (Bradley and Deavers 1980). For instance, the larger-bodied *Eptesicusfuscus* (Beauvois, 1796) is more likely to fly at low temperatures than smaller *Myotis* species (Klüg-Baerwald et al. 2016). Surprisingly, *Miniopterusschreibersii* was not as active, despite having a similar body size and using the same roost (Parnicite). This was notable given its much larger colony size of 53,822 individuals, compared to only 354 individuals of *Myotiscapaccinii*. Given that Ivanova Voda likely represents the highest known hibernaculum for *Myotiscapaccinii*, this could potentially explain the elevated activity levels observed there (Aihartza and Aizpurua 2023). Generally, the species prefers warmer roosts with high humidity, situated well below 1000 metres above sea level (Pandurska and Beshkov 1998), which once again supports the proposed unsuitability of Ivanova Voda as an explanation of the high activity levels.

Differences in the amount of fat reserves are known to influence activity levels, with fatter bats generally being more active (Czenze et al. 2017). By spending less time in torpor and maintaining a higher body temperature during this state, individuals with ample energy reserves can minimise the physiological costs associated with hibernation (Wojciechowski et al. 2007). It is likely that as an adaptation to the harsher winter conditions, the bats in Ivanova Voda enter the winter season with more sufficient fat reserves than those in Parnicite, which may contribute to their higher activity levels. Additionally, larger colonies benefit from social thermoregulation, where active bats can stimulate arousal in torpid individuals within the same cluster triggering synchronised arousal (Czenze et al. 2013). This could explain the nights with peaks in activity observed in both roosts. Further research is needed to understand the impact of fat reserves and social thermoregulation on their behaviour.

The colony at Balabanovata cave did not emerge from the cave throughout the entire winter (at least for 3 months). Despite this during our survey in the cave, we observed some minor shifts in the clustering structures and locations, suggesting some degree of activity. As an addition to our study, we placed a guano trap (as in Toshkova et al. (2023)) beneath the largest bat cluster to monitor faecal accumulation as an indicator of feeding/digestion activity. The absence of faecal buildup further supports the conclusion that the *M.myotis*/*blythii* colony was largely inactive, despite these subtle positional changes. Such prolonged torpor provides significant energetic advantages (Geiser 2004) and may also confer additional benefits, including reduced exposure to predators and has even been linked to longevity and telomere elongation (Wilkinson and South 2002, Geiser and Brigham 2012, Power et al. 2023).

The colonies of *Myotismyotis* and *Myotisblythii* at both Ivanova Voda and Balabanovata caves are affected by the WND fungus. Considering the similarities between the two roosts, we were expecting similar levels of activity; however, we recorded acoustic activity of these species only in Ivanova Voda. During our post-hibernation survey, we observed that bats in Balabanovata cave showed more visible signs of fungal infection. This may correspond to the lower levels of activity at this site, potentially leading to reduced grooming behaviour and less frequent activation of the immune system. Additionally, the strong genetic differentiation of the WND fungus population observed between the two sites (Zhelyazkova et al. 2024) may contribute to the bats' varying responses to the fungus and highlight a potential direction for future research. Of note, we recently described histopathological changes in the wing membranes in these populations caused by skin mites, *Psorergatoideskerivoulae* (Fain, 1959). The mite infestation may represent an additional stress factor for these populations and the importance of the wing membrane during the winter period emphasises the need for further research (Toshkova et al. 2022).

Importantly, we have been observing unusual mortality events during the winter monitoring exclusively in Ivanova Voda, Parnicite and Devetashkata caves (Petrov 2015, Toshkova and Deleva 2022), with no such occurrences in Balabanovata. In the winters of 2012 and 2022, we documented what are likely the highest known mortality rates recorded at European bat hibernacula (Fritze and Puechmaille 2018, Toshkova and Deleva 2022). Although the exact reasons remain unidentified, these observations may indicate differences in the suitability of roost conditions for optimal hibernation between these sites. Moreover, such mortality events are likely to become more frequent as extreme weather events and climate-induced shifts in seasonal timing increase. This highlights the importance of monitoring winter bat activity and assessing site-specific survival rates to better understand how roosting conditions and environmental pressures affect bat populations.

Overall, bat activity followed the typical circadian patterns, with peaks occurring around sunset. Similarly, other studies using both acoustic surveys and temperature-sensitive radio transmitters have shown a strong bias towards nocturnal activity (Park et al. 2000, Hope and Jones 2013). Thе low count of feeding buzzes, observed exclusively at one site (Ivanova Voda), suggests that bats likely do not use the roost vicinity for foraging during the winter. Instead, it is plausible that they relocate to other, more favourable foraging habitats, possibly at lower elevations, where food resources may be more abundant or suitable for their needs. Furthermore, our study lacks data on insect availability in the area during winter. Although some research has suggested that bats in areas with long, harsh winters may exploit high-quality foraging habitats near their hibernacula (Frick et al. 2023), this was tested using UV lights near the hibernacula. This indeed increased bats' foraging activity around the artificially created insect prey patches. However, the study was conducted during autumn swarming and spring emergence, leaving winter foraging behaviour largely unconfirmed. Additionally, it remains unclear whether winter foraging activity is primarily driven by a critical need amongst weaker individuals (Zahn and Kriner 2016) or if it represents a normal aspect of hibernators’ behaviour.

Previous research has shown that *Miniopterusschreibersii* from another hibernaculum actively feeds on diurnal insects during winter (Toshkova et al. 2023). This suggests that the lower activity levels of the species in Parnicite cave may be due to daytime foraging as an adaptive winter strategy. This likely reduces their reliance on echolocation calls and potentially indicating a greater dependence on vision during daylight hours, which could explain the low acoustic recordings. Similarly, McGowan and Kloepper (2020) hypothesised that bats dynamically rely on vision during daytime and also around cave entrances. Additionally, the presence of a resident Tawny owl, *Strixaluco* (Linnaeus, 1758), in the studied roost might influence this behaviour, potentially affecting the timing of their activity as a means of avoiding predation. However, studies on other bat species, such as the little brown bat (*Myotislucifugus*, Le Conte, 1831) and serotine bats (*Eptesicusserotinus*, Schreber, 1774), suggest that simulated owl presence at the roost entrance does not affect their departure time (Kalcounis and Brigham 1994, Petrželková and Zukal 2001, Petrželková and Zukal 2003). Further research is needed to understand the role of predation pressure during the bat hibernation period, as this factor could be an important driver of their winter behaviour. Additionally, it is plausible that the low detection of calls from the large *Miniopterusschreibersii* colony in Parnicite cave may be attributed to the use of silence as a means of avoiding acoustic interference. Similar to *Eptesicusfuscus*, which reduces vocalisation when flying with conspecifics to prevent signal jamming (Chiu et al. 2008), *Miniopterusschreibersii* may remain silent more often in densely populated roosts. This behaviour may account for the reduced number of detected calls, despite the large colony size, supporting that the bats may rely more on passive listening when flying in close proximity to one another.

The detector's location at the entrances and the variation in echolocation frequencies between bat species likely contribute to differences in their observed activity levels. In general, species that emit low-intensity calls with higher frequencies are less detectable than those using high-intensity, lower-frequency calls (Lawrence and Simmons 1982). Higher-frequency sounds attenuate more rapidly, resulting in the under-representation of these species in acoustic surveys. This issue is particularly significant for Devetashkata cave, where the roosting species exhibit a wide range of acoustic parameters. Additionally, the detector used at this site had a microphone with reduced sensitivity to high frequencies, amplifying this bias (Adams et al. 2012) and leading to a disproportionate detection of *Nyctalusnoctula* compared to other species in the cave. Unfortunately, we were also unable to collect acoustic data from this roost for the entire study period because the detector was stolen early in the survey. Despite this, we chose to report the presence of activity in this roost, as there is no available information on the presence of winter bat activity there. Given the above limitations, including the reduced microphone sensitivity at the Devetashkata site, we excluded it from further comparisons with the other sites. Furthermore, this roost has multiple entrances that bats may use during emergence, meaning our dataset captured activity from only one of these exits. Parnicite and Balabanovata Caves each have two entrances. However, it is unlikely that bats in Parnicite are using the unmonitored entrance, as they would need to fly more than 2 km through the cave to emerge from it. In Balabanovata, the second entrance is positioned close enough to the main entrance that a single detector could effectively monitor both.

#### Temperature dynamics and their influence on bat activity

As hypothesised, the temperature dynamics inside the roosting sites show that the locations where bats select to hibernate maintain stable internal conditions, which is crucial for their hibernation. Most of the study sites, including Balabanovata, Parnicite and Devetashkata caves, exhibited low R-squared values (below 0.1), indicating minimal correlation between external and internal temperatures. This suggests that these caves effectively buffer against environmental fluctuations. In contrast, Ivanova Voda cave showed a more pronounced influence of external temperature variations on the internal conditions. This cave also exhibited the highest levels of bat activity, which may be attributed to the considerable impact of external temperature on the internal conditions of the roost. The varying length and depth of the roosts are also factors that contributes to the internal temperature stability. While we were unable to collect data on humidity variation at the roosting sites, humidity is known to play a key role in winter activity patterns. Dry conditions tend to lead to more frequent arousals due to increased evaporative water loss, which may trigger the need for hydration (Ben-Hamo et al. 2013). Since all of our study sites are humid caves with running water, a future comparison of dry versus humid hibernacula could provide valuable insights.

Temperature at sunset has long been recognised as a key predictor of winter bat activity, with warmer conditions generally increasing bat activity, although the temperature threshold for this effect varies across species (Andersen et al. 2023). Our findings support this, showing that rising ambient temperatures positively influenced bat activity. Specifically, at both Ivanova Voda and Parnicite caves, external temperature was a strong positive predictor of bat activity, suggesting that warmer conditions promote higher activity levels. This aligns with our hypothesis that fluctuations in ambient temperature would affect bat activity patterns. In contrast, no correlation was found between internal temperatures and bat activity at Ivanova Voda, while at Parnicite, internal temperature had a surprisingly significant negative effect. We found this result difficult to interpret, but it is important to note that the internal temperature at Parnicite is relatively stable (7.86°C ± 0.22°C). Given these minimal fluctuations, it is questionable whether such small temperature changes could have a biologically significant impact on bat activity patterns. Similarly, Halsall et al. (2012) found no correlation between roost temperature and torpor bout length, suggesting that wider ranges in roost temperatures may make detecting patterns in activity easier, as bats are likely to respond more noticeably to broader temperature shifts. Our data reflect only external bat activity, lacking information on in-roost patterns, which are more likely influenced by internal temperature conditions. Additionally, the zero-inflation models for both caves found no significant predictors for the absence of bat activity, as neither external nor internal temperatures were significantly associated with the likelihood of observing bat activity. This suggests that factors beyond temperature, such as arousal patterns, play a more significant role in determining bat inactivity.

Our analysis of historical temperature trends reveals a substantial decline in the number of days with temperatures below 0°C, alongside a projected increase in days surpassing 5°C. These shifts reflect an overarching trend towards milder climatic conditions, which may extend the active periods of hibernating bat species both within the study areas and across Bulgaria. These findings, spanning over seven decades, are statistically significant and align with broader climatological studies in the region, suggesting the shift of the climate in the region towards higher temperatures in the winter periods and lower precipitation (Marinova and Bocheva 2023). Such changes may disrupt the thermal stability of roost sites and alter seasonal behaviour within bat populations, warranting further investigation into the ecological impacts of climate change, especially the potential mismatch between bat activity and prey availability (Festa et al. 2022).

The effective assessment and management of climate change impacts on biodiversity rely heavily on the presence and strategic collection of high-resolution local data, as well as its integration into global models. Global warming is expected to bring more frequent extreme temperature fluctuations, posing additional challenges. While some threats, such as cave flooding, lie beyond the scope of conservation efforts, it is crucial to recognise and address these as significant risk factors for underground hibernacula. Simultaneously, this underscores the need to proactively mitigate other controllable threats to better protect the fragile balance of our increasingly vulnerable environment.

## Supplementary Material

E61C4704-AE23-5BE8-8829-35525F62807D 10.3897/BDJ.13.e141801.suppl1 Supplementary material 1 Supplementary Material 1 Data type bat activity data Brief description The dataset captures detailed observations of bat activity across four roost sites. Each entry includes the cave location, the exact date and time of the observation and the recorded temperature at that moment. Bat activity is classified by species and behaviour, with specific indicators for social calls and feeding buzzes. If social calls were detected during a recording, the 'social_call' column is marked TRUE; similarly, if feeding buzzes were recorded, the 'feeding_buzz' column is marked TRUE. File: oo_1172177.xlsx https://binary.pensoft.net/file/1172177 Nia Toshkova

FDD59295-5B18-5CB0-95DC-024085A6925F 10.3897/BDJ.13.e141801.suppl2 Supplementary material 2 Supplementary Materials 2 Data type temperature data Brief description This dataset records average daily external and internal temperatures at bat colony sites in four caves (Balabanovata, Parnicite, Ivanova and Devetashkata), with each entry including the date and temperature readings for each location. File: oo_1172184.xlsx https://binary.pensoft.net/file/1172184 Nia Toshkova

310B7B1E-6A45-5483-8260-1B0889DEB739 10.3897/BDJ.13.e141801.suppl3 Supplementary material 3 Supplementary Materials 3 Data type historical temperature data Brief description This dataset contains historical records of average daily external temperatures for January, February and March from 1950 to 2022 for four caves (Ivanova, Balabanova, Parnicite and Devetashkata), organised by month and year and sourced from the nearest available temperature nodes. File: oo_1172189.xlsx https://binary.pensoft.net/file/1172189 Nia Toshkova

## Figures and Tables

**Table 1.** Study site information. The first three columns represent the site location and altitude above sea level. The fourth column shows the number of species present year-round, based on historical data. The fifth column indicates the number of hibernating bat species. The BP column represents the Biotic Potential index, while the BV column represents Biotic Vulnerability. The last column indicates the presence of WND at the site.

**Roost**	**Lat (N)**	**Lon (E)**	**Alt (m)**	Roost length (m)/ depth (m)	**Species YR**	**Species Hibernation**	**BP index**	**BV index**	**WND**
Devetashkata	43.23	24.88	133	2442 /0	17	3	1	A	-
Balabanovata	43.13	23.04	1285	3351/126	12	1	1	C	+
Ivanova Voda	41.89	24.88	1325	695/133	8	3	1	C	+
Parnicite	43.2	24.41	232	2500/32	5	3	1	B	-

**Table 2.** Hibernacula census data and acoustic survey details for each study site.

**Roosts**	№ **оf species**	**Total № оf ind. 2022**	**Start Survey** **(dd/mm/yyyy)**	**End Survey** **(dd/mm/yyyy)**	**Days of recordings**	**Total bat call sequences (5 sec)/nights of activity**
Balabanovata	1	2500	15/01/2022	31/03/2022	75	25/1
Ivanova Voda	2	7000	22/01/2022	28/03/2022	65	10568/29
Parnicite	3	56640	26/01/2022	15/03/2022	47	1190/7
Devetashkata	3	4349*	26/01/2022	04/02/2022	9	171/9

**Table 3.** Number of bat passes in each hibernaculum for all identified bat species and phonic groups. TBP indicates the total percentage (%) of bat passes per species and (n) represents the number of days recorded per roost. Species abbreviations: Msch – *Miniopterusschreibersii*; Myotis – *Myotis* sp.; Mcap – *Myotiscapaccinii*; Mmyo/bly – *Myotismyotis*/*blythii*; Nnoc – *Nyctalusnoctula*; Plecotus – *Plecotusauritus*/*austriacus*; Pkuh/Pnat – *Pipistrelluskuhlii*/*nathusii*; Ppip – *Pipistrelluspipistrellus*; Rhfer – *Rhinolophusferrumequinum*; Rhip – *Rhinolophushipposideros*; Hsavi – *Hypsugosavii*; Eser – *Eptesicusserotinus*.

**Bat species and acoustic groups**	**Balabanova dupka (n = 75)**	**Ivanova Voda (n = 65)**	**Parnicite (n = 47)**	**Devetashkata (n = 9)**	**Total**	**TBP (%)**
Msch	0	124	90	0	214	1.8
Myotis	0	1140	0	0	1140	9.5
Mcap	0	7313	1012	3	8328	69.7
Mmyo/bly	0	1959	0	1	1960	16.4
Nnoc	0	2	0	132	134	1.1
Plecotus	0	2	0	0	2	0.02
Pkuh/Pnat	0	0	0	21	21	0.2
Ppip	0	3	0	1	4	0.03
Rhfer	0	8	88	12	108	0.9
Rhip	25	3	0	0	28	0.2
Hsavi	0	0	0	1	1	0.008
Eser	0	14	0	0	14	0.1
**Total**	25	10568	1190	171	11954	

**Table 4.** Summary statistics of the external temperature (°C) for each site throughout the study period.

**Site**	**Mean**	**Median**	**SD**	**Min**	**Max**	**Range**
Devetashkata external	4.46	3.93	4.49	-4.11	17.4	21.5
Devetashkata internal	13	13	0.306	12.4	13.7	1.26
Parnicite external	3.92	3.35	4.42	-4.42	16.3	20.7
Parnicite internal	8.02	7.99	0.319	7.48	8.74	1.26
Ivanova Voda external	-3.25	-3.59	4.98	-13.3	7.2	20.5
Ivanova Voda internal	3.21	3.05	0.483	2.48	4.13	1.64
Balabanovata external	-2.79	-3.68	5.20	-13.4	7.22	20.6
Balabanovata internal	4.26	4.22	0.289	3.72	4.91	1.19

**Table 5.** Number of days in each location when the external temperature at sunset and the mean daily temperature were above certain thresholds, based on the data from January, February and March 2022.

	**Sunset temperatures**			**Mean daily temperatures**		
**Location**	**Days > 0°C**	**Days > 5°C**	**Days > 10°C**	**Days > 0°C**	**Days > 5°C**	**Days > 10°C**
Balabanovata	28	6	0	28	6	0
Ivanova Voda	27	4	0	27	4	0
Parnicite	76	28	8	68	31	5
Devetashkata	78	35	9	75	34	5
